# Alfalfa transcriptomic responses to the field pathobiome

**DOI:** 10.1111/plb.70021

**Published:** 2025-04-15

**Authors:** L. G. Nemchinov, B. M. Irish, S. Grinstead, O. A. Postnikova

**Affiliations:** ^1^ USDA‐ARS, Beltsville Agricultural Research Center Molecular Plant Pathology Laboratory Beltsville Maryland USA; ^2^ USDA‐ARS Plant Germplasm Introduction and Testing Research Unit Prosser Washington USA; ^3^ USDA‐ARS, Beltsville Agricultural Research Center Animal Biosciences and Biotechnology Laboratory Beltsville Maryland USA

**Keywords:** field host genomics, *Medicago sativa* L., pathobiome, transcriptomics

## Abstract

- The pathobiome is a comprehensive biotic environment that includes a community of all disease‐causing organisms within the plant, defining their mutual interactions and the resultant effects on plant health. The concept and understanding of alfalfa pathobiome and its impact on host fitness in natural ecosystems remain largely unexplored.
- We have previously reported on the diverse composition of the alfalfa pathobiome in the field production environment. In this study, using modern transcriptomics tools combined with computational analyses, we applied a novel ‘field host genomics’ approach to survey gene expression changes in visually healthy and diseased plants collected from commercial alfalfa fields.
- As a result of this work, genes and pathways involved in alfalfa responses to a diverse field pathobiome were identified and the genetic basis of the crop's resistance to multi‐pathogenic infections was proposed.
- In addition to offering genetic insights into host resistance to multi‐pathogenic infections in natural ecosystems, this strategy can facilitate identification of plants with tolerant genotypes adapted to field pathobiome, followed by their application in alfalfa breeding programs.

## Graphical abstract

A novel ‘field host genomics approach’ allowed identification of genes and pathways involved in alfalfa responses to a diverse field pathobiome.

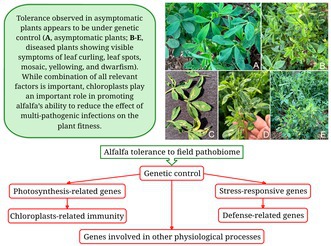

## INTRODUCTION

With the development of modern high‐throughput sequencing approaches, the “one microbe—one disease” concept is being largely re‐evaluated and replaced with the principle of a “pathobiome”. A pathobiome is a comprehensive biotic environment that includes a diverse community of all disease‐causing organisms within the plant (Vayssier‐Taussat *et al*. 2014; Bass *et al*. 2019; Mannaa & Seo 2021). While numerous individual pathogens and diseases they cause in alfalfa (*Medicago sativa* L.), the most extensively cultivated forage legume in the world, were described in detail, the concept and understanding of the crop's pathobiome remain largely unexplored. We have recently demonstrated that alfalfa pathobiome represents a sophisticated habitat which encompasses at least 20–30 potential pathogens in individual plants (Nemchinov *et al*. 2023a). In theory, such a complex multi‐pathogenic environment may not only affect the behaviour of all coinfecting organisms, their accumulation in the host, virulence, disease aetiology, and epidemiology, but also impact host fitness, prompting unique genetic responses to the collective pathobiome. These responses could be assessed for their roles in resistance to multiple biological stressors in a natural field environment, contrary to the standard practice of evaluating a single disease‐causing agent under controlled experimental conditions. To the best of our knowledge, this “field host genomics” approach is missing in the literature and has yet been undertaken in alfalfa or any other crop. Moreover, revealing genetic basis of host resistance to multi‐pathogenic infections can improve our understanding of pathogenicity and further accelerate breeding programs.

In this work, we applied the proposed strategy to estimate host genetic responses to a collective pathobiome in commercial alfalfa fields. Since in the natural environment all field plants are impacted by various organisms to some degree, it is not possible to obtain *de facto* uninfected control samples for transcriptomic analysis. To overcome this issue in experiments described herein, visually asymptomatic plants with healthy appearance were selected from the same fields where symptomatic/diseased plants were collected. While healthy‐looking plants may contain various microbial communities of commensal, mutualistic or latent nature, organisms inhabiting them do not result in any symptoms and disease development with minimal to no damage to the host, if any (Sinclair 1991). Importantly, these non‐affected plants collected from the same fields as diseased samples, may potentially be considered tolerant or resistant to multi‐pathogenic infections (Hull 2014). Using modern transcriptomics tools combined with computational analyses, we assessed gene expression patterns in symptomless alfalfa plants and compared these to samples displaying obvious symptomatology and disease damage. As a result of this work, genes and pathways involved in alfalfa responses to a diverse field pathobiome were identified and the genetic basis of resistance to multi‐pathogenic infections was proposed.

## METHODS

### Plant material and sample collection

Samples were collected with owners' permission from five commercial alfalfa fields in Benton and Yakima Counties, Washington State, USA, during mid‐July 2024 (Table S1). Benton and Yakima are large agricultural counties in the shrub‐steppe dessert of south‐central Washington. Alfalfa samples were collected in and around the towns of Prosser and Grandview, WA with an approximate elevation of 150 m on well‐drained, moderately deep, medium‐textured soils.

Five leaves, including all three leaflets and petiolules, were collected from each of five different asymptomatic and, separately, from five symptomatic plants of similar stage of development in five individual fields. The materials from five asymptomatic and, separately, from five symptomatic plants collected from each of the five fields were pooled for nucleic acid extraction to allow more efficient and comprehensive screening of a large population of plants (i.e. field). This resulted in two pooled samples from each field and 10 samples in total, that is, five biological replications for each sample type (asymptomatic and symptomatic). Asymptomatic samples were provisionally designated as “healthy” (H) and symptomatic “diseased” (D). Sampling was done in a zigzag or “W” pattern to include representative samples across the entire field. After collection, samples were immediately placed on dry ice and subsequently stored at −80°C until extraction.

### Nucleic acid extraction

Pooled leaves from symptomatic and asymptomatic plants were pulverized in liquid nitrogen and used for RNA and DNA extractions. Total RNA extractions were performed with Qiagen's RNeasy Mini Kit (50) according to manufacturer's directions (Qiagen, Germantown MD, USA), and genomic DNA extractions were done using Qiagen's DNeasy PowerSoil Pro Kit (50) per the manufacturer's instructions. In our hands, DNeasy PowerSoil Pro Kit worked better than any other kit for DNA extraction from plant tissues.

### RNA and amplicon sequencing

Library preparation and Illumina RNA sequencing were performed by Novogene (Novogene, Sacramento, CA, USA). Messenger RNA was purified from total RNA using poly‐T oligo‐attached magnetic beads. The cDNA library was prepared using ABclonal Fast RNA‐seq Lib Prep Kit v. 2 (ABclonal Technology, Wobum MA, USA). The library was checked with a Qubit and real‐time PCR for quantification, and a bioanalyzer for size distribution detection. Quantified libraries were sequenced on Illumina NovaSeq X Plus platform (PE150; 12 Gb or 40 million reads per sample).

The 16S and ITS (Internal Transcribed Spacer) library preparation for ribosomal RNA (rRNA) sequencing was carried out using an ABclonal Rapid Plus DNA Lib Prep Kit (Abclonal, Wobrun, MA, USA). Libraries were sequenced on Illumina NovaSeq 6000 platform (PE250, 50 K raw tags per sample). To discriminate from chloroplast DNA, primers targeted amplification of the DNA encoding V5‐V7 region of the bacterial 16S rRNA gene: 799F, 5′ AACMGGATTAGATACCCKG 3′ and 1193R, 5′ ACGTCATCCCCACCTTCC 3′, with Illumina adapters. Primers used for amplification of the fungal internal transcribed spacer (ITS) region were ITS1F (F) 5′ CTTGGTCATTTAGAGGAAGTAA 3′ and ITS2R (R) 5′ GCTGCGTTCTTCATCGATGC 3′ (Nemchinov *et al*. 2023b).

### Bioinformatics analysis

Bioinformatics analysis was performed both in‐house and as a part of the RNA and Amplicon sequencing service provided by Novogene. Novogene's workflow for bioinformatic analysis is shown in Figure S1. Reads were assembled with StringTie (Pertea *et al*. 2015) and quantified with featureCounts (Liao *et al*. 2014). For differential analysis, DESeq2 and edgeR were used (Love *et al*. 2014; Robinson *et al*. 2010, respectively). Gene expression was estimated by the abundance of transcripts that mapped to the alfalfa reference genome (Chen *et al*. 2020) genome. FPKM values (Fragments Per Kilobase of transcript sequence per Millions base pairs sequenced) (Trapnell *et al*. 2010) were used for estimating gene expression levels, taking into consideration the effect of both sequencing depth and gene length on counting of fragments (Mortazavi *et al*. 2008).

Alignment to the reference was performed with HISAT2 software (Mortazavi *et al*. 2008). For functional analyses, including GO (Gene Ontology) Enrichment, DO (Disease Ontology) Enrichment, and KEGG (Kyoto Encyclopedia of Genes and Genomes), clusterProfiler was used (Yu *et al*. 2012). Gene Set Enrichment Analysis (GSEA) was performed using GSEA software (https://www.gsea‐msigdb.org/gsea/index.jsp). Principal components analysis (PCA) was used to evaluate intra‐ and inter‐group differences (Jolliffe & Cadima 2016). The protein–protein interaction network was constructed by the searching protein interaction database STRING (https://string‐db.org). SNP/InDel Analysis was performed using the snpEff tool (Cingolani *et al*. 2012).

The QIIME analysis (Quantitative Insights Into Microbial Ecology), (Caporaso *et al*. 2010) was used to identify fungal and bacterial communities in 16S and ITS sequencing data. OTU (Operational Taxonomic Units) clustering was employed for taxonomic annotations. OTUs were analysed for relative abundance, taxonomic abundance, common and unique groups (Venn diagram, and Flower diagrams), and phylogenetic relationships. Alpha and Beta Diversity analyses were performed to analyse composition of microbial communities in different samples.

Bioinformatics pipeline for virus identification was used essentially as described in Nemchinov *et al*. (2023a).

## RESULTS

### Pathogen identification and field pathobiome of symptomatic and asymptomatic alfalfa plants

**Fig. 1:**
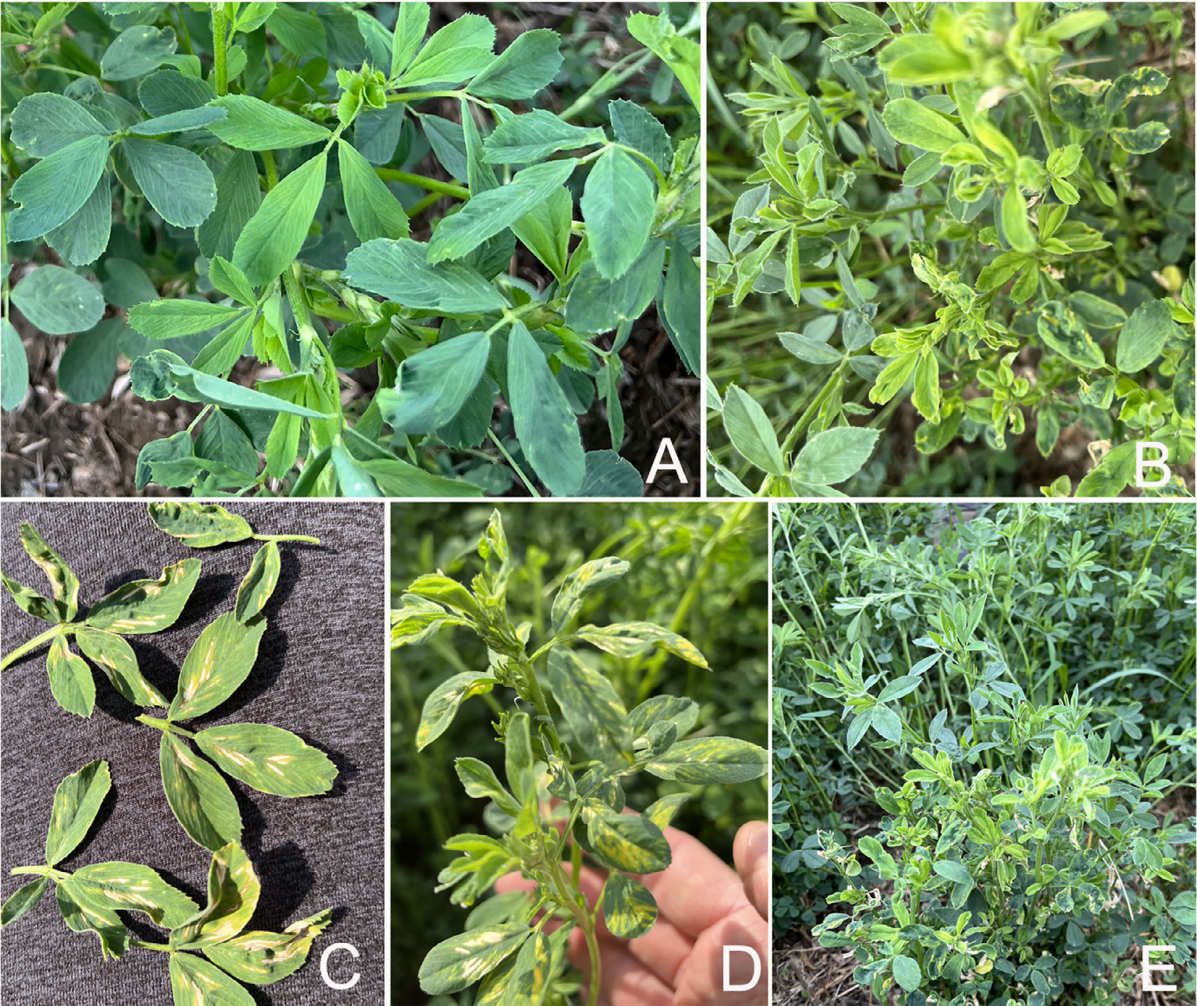
Phenotypes of asymptomatic (A) and symptomatic (B–E) alfalfa plants. (B), leaf curling and malformation. (C) Leaf spots and necrotic lesions. (D) Yellowing mosaic symptoms, likely AMV. (E) shoot proliferation and stunting.

The complexity of the alfalfa field pathobiome was evaluated with emphasis on the composition of viral, bacterial, and fungal communities. Diseased plants displayed a variety of symptoms including leaf curling, yellowing, mosaic, mottling, chlorosis, local lesions, dwarfing, leaf spots, wilts, blights, anthracnose, crown and stem rot, while “healthy” plants did not exhibit any obvious symptomatology (Fig. 1).

#### Viruses

**Fig. 2:**
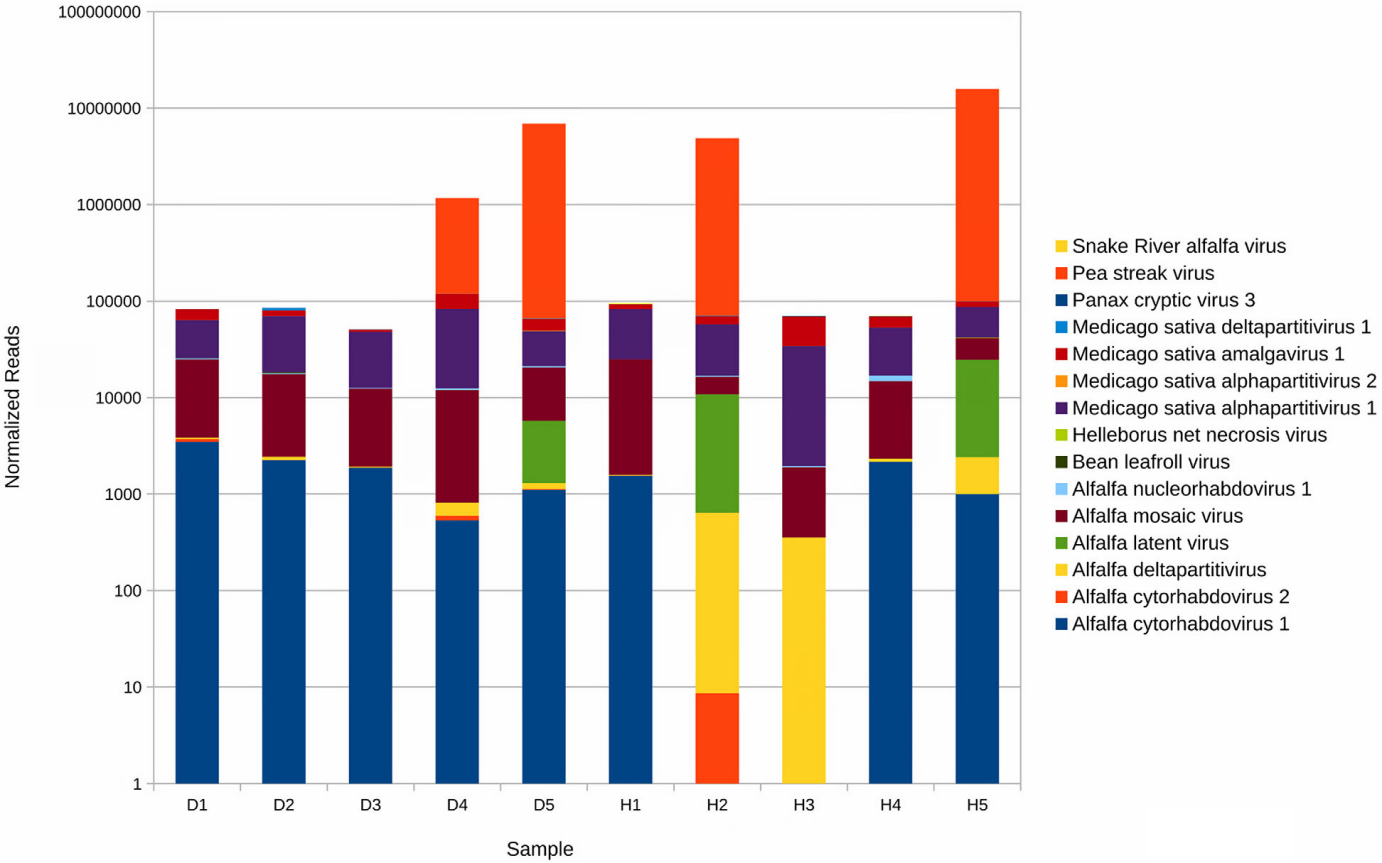
Composition and relative abundance of the alfalfa (*Medicago sativa* L.) virome in 50 symptomatic (D) and asymptomatic (H) plant samples based on the results of RNA‐sequencing.

The total number of plant viral reads for all diseased plants was 7,710,052, and for all healthy‐appearing plants was 19,407.753. These numbers were dominated by pea streak virus (PeSV), (7,333,345 and 19,028,984 reads, respectively) (Table S2). Although PeSV is usually symptomless in alfalfa, it is transmissible to susceptible legume hosts, like pea and lentil crops (Larsen 2015). All other plant viruses, excluding pea streak virus, had 376,707 and 378,769 reads, respectively. Therefore, both asymptomatic and symptomatic plants were infected to an approximately similar extent (Fig. 2). The predominant species were PeSV, Medicago sativa alphapartitivirus 1, alfalfa mosaic virus (AMV), Snake River alfalfa virus (SRAV), alfalfa rhabdoviruses, alfalfa latent virus (ALV), and bean leafroll virus (BLRV)—all present in both asymptomatic and symptomatic plants. As some of these viruses, especially AMV, may cause distinct symptomatology, one could suggest that healthy‐appearing plants collected from the same field as diseased plants exhibit tolerance to the infection, possibly due to genetic heterogeneity, resistance to aphids, or absence of seed transmission.

#### Bacteria

As a result of the 16S rRNA sequencing, 1,222,165 clean bacterial reads were obtained that were annotated to OTUs, followed by their classification at the level of kingdom, phyla, class, order, family, genus, and species. Bacterial species identified across the two groups are shown in Table S3. Composition and diversity of the identified OTUs were assessed by relative and taxonomic abundance analyses, and by alpha and beta diversity index values of amplicon sequence data.

**Fig. 3:**
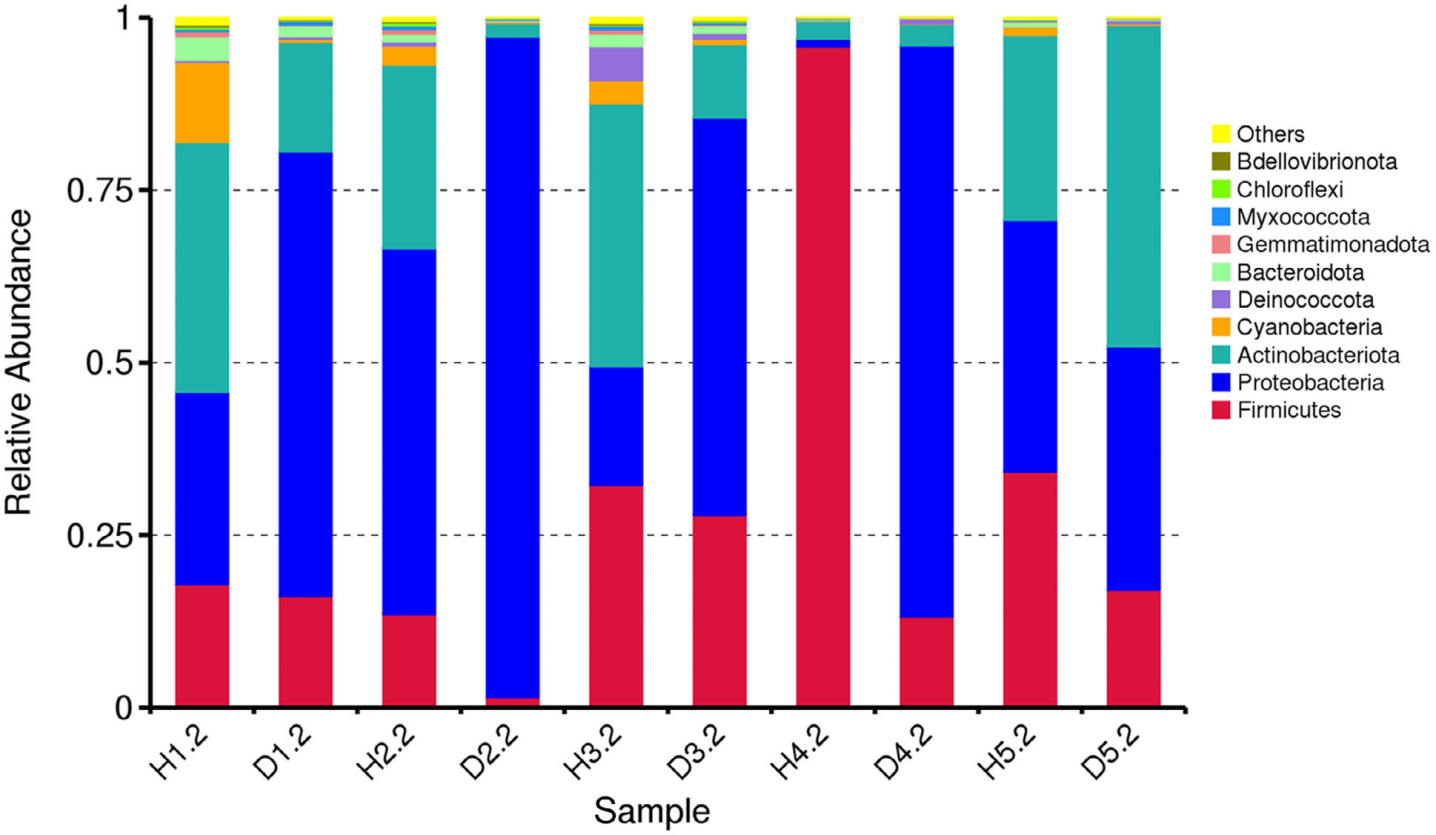
Composition and relative abundance of the alfalfa (*Medicago sativa* L.) bacteriome at the phyla level based on the results of 16S V5‐V7 amplicon sequencing.

Predominant relative abundance was registered for the populations of bacteria belonging to phyla *Actinobacteriota*, *Proteobacteria*, and *Bacillota* (*Firmicutes*) (Fig. 3). While most actinobacteria and some firmicutes are beneficial to plants, many species in the phylum *Proteobacteria* are pathogenic. On average, distribution of relative abundance was comparable between symptomatic and asymptomatic plants. The taxonomic abundance cluster heatmap showed that diseased samples had a noticeable presence of several pathogenic species (*Pseudomonas viridiflava*, *Agrobacterium rubi*, *Acidovorax* sp.) (Figure S2) absent in asymptomatic plants, which were often infected with known beneficial bacteria. Nevertheless, similar to our previous study on the alfalfa pathobiome (Nemchinov *et al*. 2023b), the occurrence of pathogenic bacteria in symptomatic alfalfa field samples was not extensive.

Alpha diversity analysis performed to assess richness of microbial communities in each group, showed greater diversity of bacterial species in asymptomatic plants (Figure S3). Meanwhile, the beta‐diversity heatmap indicated that dissimilarity coefficients between bacterial communities of symptomatic and asymptomatic plants originated from the same fields were generally low (Figure S4), thus indicating an overall low species diversity.

Therefore, analogous to the virome, 16S rRNA sequencing demonstrated that the bacteriome of asymptomatic and symptomatic alfalfa plants in the field was comparable.

#### Fungi

**Fig. 4:**
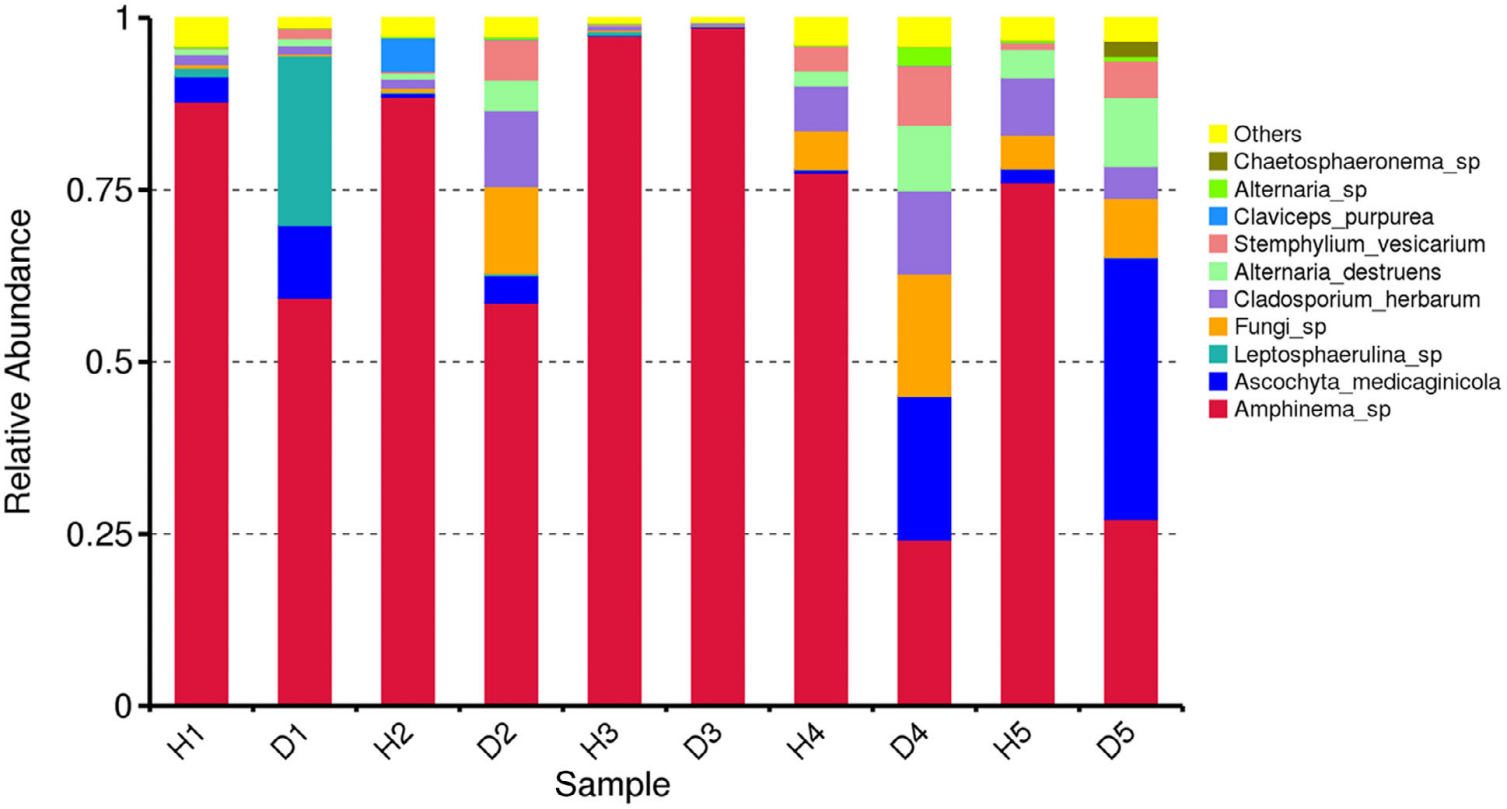
Composition and relative abundance of fungal species in alfalfa (*Medicago sativa* L.). field samples based on the results of ITS1‐2 amplicon sequencing.

In total, 972,315 clean combined reads were obtained by the ITS sequencing. Sequences were annotated to the level of kingdom, phyla, class, order, family, genus, and species. Species identified in all samples are shown in Table S3. Relative abundance registered at the species level showed the presence of pathogenic *Stemphylium* sp., *Ascochyta medicaginicola*, *Alternaria* sp., *Cladosporium herbarum*, *Leptosphaerulina* sp., *Fusarium* sp., and others in symptomatic and asymptomatic plants (Fig. 4). All samples contained reads of *Amphinema* sp., corticioid fungi in the family Atheliaceae, known as wood‐rotting species. The taxonomic cluster heatmap showed the abundance of the species in each sample, confirming the output of relative abundance distribution (Figure S5). Alpha diversity analysis indicated a greater diversity of observed species in asymptomatic samples (Figure S6), while beta diversity analysis suggested, with few exceptions, an overall low level of dissimilarities between samples originating from the same fields (Figure S7). Once again, although diseased plants were infected with several known fungal pathogens, plants displaying no visual symptoms were not pathogen‐free.

### Gene expression in symptomatic versus asymptomatic alfalfa plants

#### Metrics of RNA‐seq data

Metrics of RNA‐seq data are shown in Table S1. A total of 837,870,186 clean paired‐end reads were generated from 10 cDNA libraries, thus averaging 83,787,019 paired‐end or 41,893,509.3 single‐reads per library. The alignment rate for each library ranged between 69% and ~87% mapped to the reference alfalfa genome (Chen *et al*. 2020). The RNA sequencing error rate was low, 0.01 (1 in 10,000 bases), indicating high‐quality sequencing acceptable for the experiment. Overall, the data obtained were sufficient for gene expression profiling.

#### Differentially expressed genes (DEGs) in symptomatic versus asymptomatic alfalfa plants

**Fig. 5:**
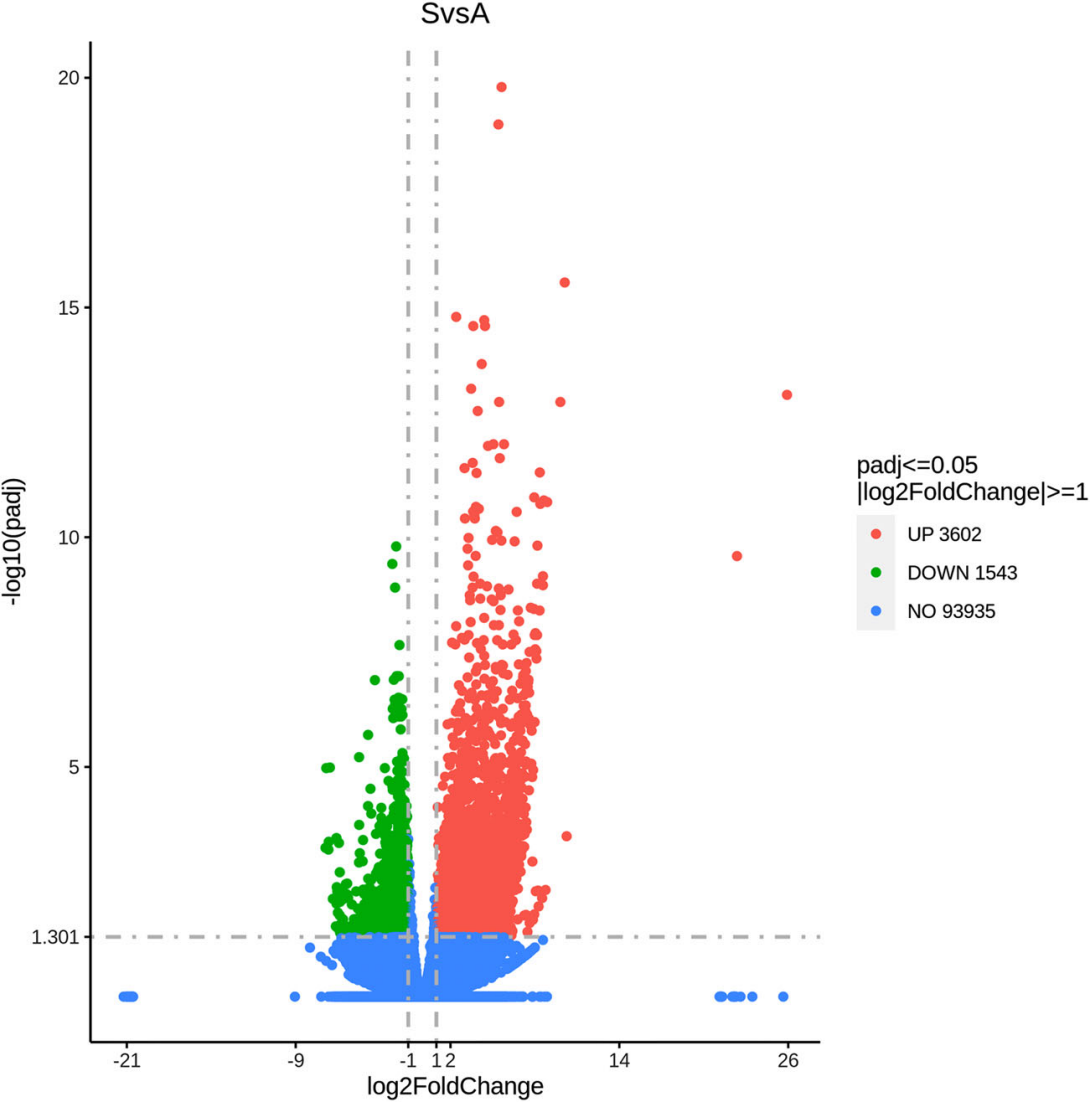
Volcano plot showing the overall distribution of DEGs. The *x*‐axis shows the fold change in gene expression between different samples, and the *y*‐axis shows the statistical significance of the differences. Red dots represent upregulated genes, and green dots represent downregulated genes. The blue dashed line indicates the threshold line for differential gene screening criteria.

Two groups were assembled to analyse differential expression in symptomatic versus asymptomatic alfalfa phenotypes: one included all “healthy” samples collected from five different fields (H1–H5), and the other—all diseased samples (D1–D5). The first group was designated ‘A’ for asymptomatic and the second group ‘S’, for symptomatic phenotypes. A summary of DEGs statistics between the S and A groups is shown in Table S1, and their overall distribution, statistical significance and fold changes is presented in Fig. 5.

The total number of upregulated DEGs in symptomatic plants was significantly higher, indicating overall higher gene activity and mRNA production levels in diseased plants. Genes involved in RNA degradation (e.g., MS_gene91185, exosome complex component RRP43); proteosome components (e.g., MS_gene058875, 26S proteasome non‐ATPase regulatory subunit 2); protein export (MS_gene0874; ATPase GET3B); disease resistance (e.g., MS_gene44944, MS_gene87339, etc.), and many other processes were upregulated in the diseased phenotypes, suggesting vigorous anti‐pathogenic responses are taking place. All individual DEGs, along with their expression values, gene IDs, and annotations are shown in Tables S4– S6. Cluster analysis was caried out to learn if genes with similar expression patterns can be grouped. Three groups of genes depicted expression changes between diseased and “healthy” plants: genes involved in photosynthetic processes, which were normally downregulated in diseased samples (Figure S8); genes encoding heat shock proteins upregulated in diseased plants; and defence‐ and pathogenesis‐related genes that had elevated expression in the symptomatic plants.

The most highly expressed genes in both phenotypes, according to the normalized read counts, were those involved in photosynthetic reactions (Tables [Link], [Link], [Link]). Genes encoding ribulose‐1,5‐bisphosphate carboxylase/oxygenase (Rubisco), that catalyses fixation of carbon from atmospheric CO_2_ (Bathellier *et al*. 2020) and participates in photorespiration, were expressed at higher levels in asymptomatic phenotypes. Via the photorespiratory pathway, Rubisco can mitigate oxidative stress, down‐regulating production of reactive oxygen species in the chloroplast during immune response and protect photosystems from photodamage (Voss *et al*. 2013; Jiang *et al*. 2023). Oxygen‐evolving enhancer proteins (PSBO), a critical part of the photosynthetic complex important for thylakoid architecture and involved in host–pathogen interactions (Kong *et al*. 2014) and light‐harvesting complex proteins, essential for plant photosynthetic machinery (Tanaka *et al*. 2010), were also highly expressed in asymptomatic phenotype versus symptomatic phenotype.

Other known key regulators of plant immune response associated with chloroplasts, such as calcium‐sensing receptor (CAS) mediating restriction of bacterial growth, gene encoding thylakoid formation protein (ThF1), mediating both PTI (pattern‐triggered immunity) and ETI (effector‐triggered immunity) responses (Kachroo *et al*. 2021), and light‐harvesting complex‐like proteins, contributing to pathogen resistance and protection of chloroplasts from oxidative stress (Hey & Grimm 2018; Liu *et al*. 2019), were expressed at higher levels in ‘A’‐phenotype.

There were many silent or uniquely expressed genes (non‐expressed or expressed at a very low level with near‐zero read counts) in both phenotypes. When a gene is silent in ‘A’‐ and expressed in ‘S’‐phenotype, this points to its activation due to the host–pathogen interactions, while when the opposite happens, it may indicate its role in mechanisms of resistance to field pathobiome.

The top ten uniquely expressed genes upregulated in the ‘S’ and silent in the ‘A’ phenotype are shown in Table 1. Among them were those encoding Kunitz trypsin inhibitor, involved in defence against pests and herbivores (Bonturi *et al*. 2022); heat shock proteins, including HSP90, mediating stress signal transduction; laccase (Lac 7) that plays important roles in plant defences (Yu *et al*. 2021); VITVI Agamous‐like MADS‐box protein, a transcriptional regulator involved in stress responses (Castelan‐Munoz *et al*. 2019), and others. These genes were also at the top of the list of highly upregulated genes, thus emphasizing their vital roles in alfalfa interactions with the field pathobiome (Xu *et al*. 2012).

**Table 1:** Top 10 uniquely expressed upregulated genes in symptomatic (‘S’) and asymptomatic (‘A’) phenotypes.

‘S’ phenotype					
gene_ID	log2FoldChange	*P*‐value	padj	gene_chr	protein and gene description
MS_gene50908	25.91497945	1.10E‐17	7.98E‐14	chr3.3	|Q41015|PIP21_PEA Kunitz‐type trypsin inhibitor‐like 1 protein OS = Pisum sativum
MS_gene39508	22.36125335	1.52E‐13	2.58E‐10	chr4.2	|P27880|HSP12_MEDSA 18.2 kDa class I heat shock protein OS = Medicago sativa
MS_gene04350	10.25462041	3.47E‐06	0.0003224	chr3.4	|P55737|HS902_ARATH Heat shock protein 90–2 OS = Arabidopsis thaliana
MS_gene048082	10.1199187	1.18E‐20	2.86E‐16	chr4.4	|Q9SR40|LAC7_ARATH Laccase‐7 OS = Arabidopsis thaliana
MS_gene38363	9.810193692	1.88E‐17	1.14E‐13	chr3.2	|Q9SND9|Y3028_ARATH Uncharacterized acetyltransferase OS = Arabidopsis thaliana
MS_gene47682	8.872085208	5.67E‐15	1.72E‐11	chr5.3	|P24076|BGIA_MOMCH Glu S.griseus protease inhibitor OS = Momordica charantia
MS_gene49154	8.758466235	0.0001264	0.0047591	chr1.3	|P51819|HSP83_IPONI Heat shock protein 83 OS = Ipomoea nil
MS_gene013756	8.632169259	5.01E‐15	1.58E‐11	chr3.3	|Q0HA25|MADS9_VITVI Agamous‐like MADS‐box protein MADS9 OS = Vitis vinifera
MS_gene04024	8.572115155	4.47E‐13	7.06E‐10	chr3.3	|Q01807|LEC2_MEDTR Truncated lectin 2 OS = Medicago truncatula
MS_gene011597	8.57085468	7.64E‐13	1.11E‐09	chr8.1	|Q9M7K4|POT5_ARATH Potassium transporter 5 OS = Arabidopsis thaliana

‘A’ phenotype					
gene_ID	log2FoldChange	*P*‐value	*p*adj	gene_chr	protein and gene description
MS_gene97260	−6.8757669	7.62E‐06	0.0005771	chr6.4	Medicago truncatula uncharacterized LOC25495897
MS_gene20930	−6.8240864	4.38E‐08	1.06E‐05	chr1.3	|PF13419: Haloacid dehalogenase‐like hydrolase
MS_gene068502	−6.6725796	8.60E‐06	0.0006287	chr4.2	|Q7XPY2|PMA1_ORYSJ Plasma membrane ATPase OS = Oryza sativa subsp. japonica
MS_gene067994	−6.6491031	5.01E‐06	0.0004256	chr8.3	Hypothetical protein MtrunA17_Chr7g0215391 [Medicago truncatula]
MS_gene21834	−6.569530738	4.22E‐08	1.03E‐05	chr5.2	|Q8GTE3|RS3A_CICAR 40S ribosomal protein S3a OS = Cicer arietinum
MS_gene87925	−6.1429706	0.0016532	0.0294615	chr6.1	|B8BJ39|KPYC1_ORYSI Pyruvate kinase 1, cytosolic OS = Oryza sativa subsp. indica
MS_gene72202	−6.0943342	0.0003328	0.0094798	chr5.1	|PF03106: WRKY DNA‐binding domain
MS_gene99515	−6.0812176	0.0001038	0.0041409	chr1.3	|Q6E593|BEBT1_PETHY Benzyl alcohol O‐benzoyltransferase OS = Petunia hybrida
MS_gene016372	−6.0392219	0.0001403	0.0051221	chr8.2	|PF00400: WD domain, G‐beta repeat
MS_gene045088	−6.0070489	0.0018772	0.0321011	chr3.4	|Q02920|NO70_SOYBN Early nodulin‐70 OS = Glycine max

Among the top ten genes silent in the ‘S’ and expressed in the ‘A’ phenotype were haloacid dehalogenase‐like hydrolase involved in stress responses (Zan *et al*. 2023); plasma membrane ATPase, participating in plant–microbe interactions (Elmore & Coaker 2011); 40S ribosomal protein S3a, multifunctional protein regulating DNA repair, apoptosis, and plant innate immune response to bacterial infection (Gao & Hardwidge 2011); DNA‐binding domain of WRKY transcription factor, known for their role in plant immunity (Table 1) (Pandey and Somssich, 2009) and others.

**Fig. 6:**
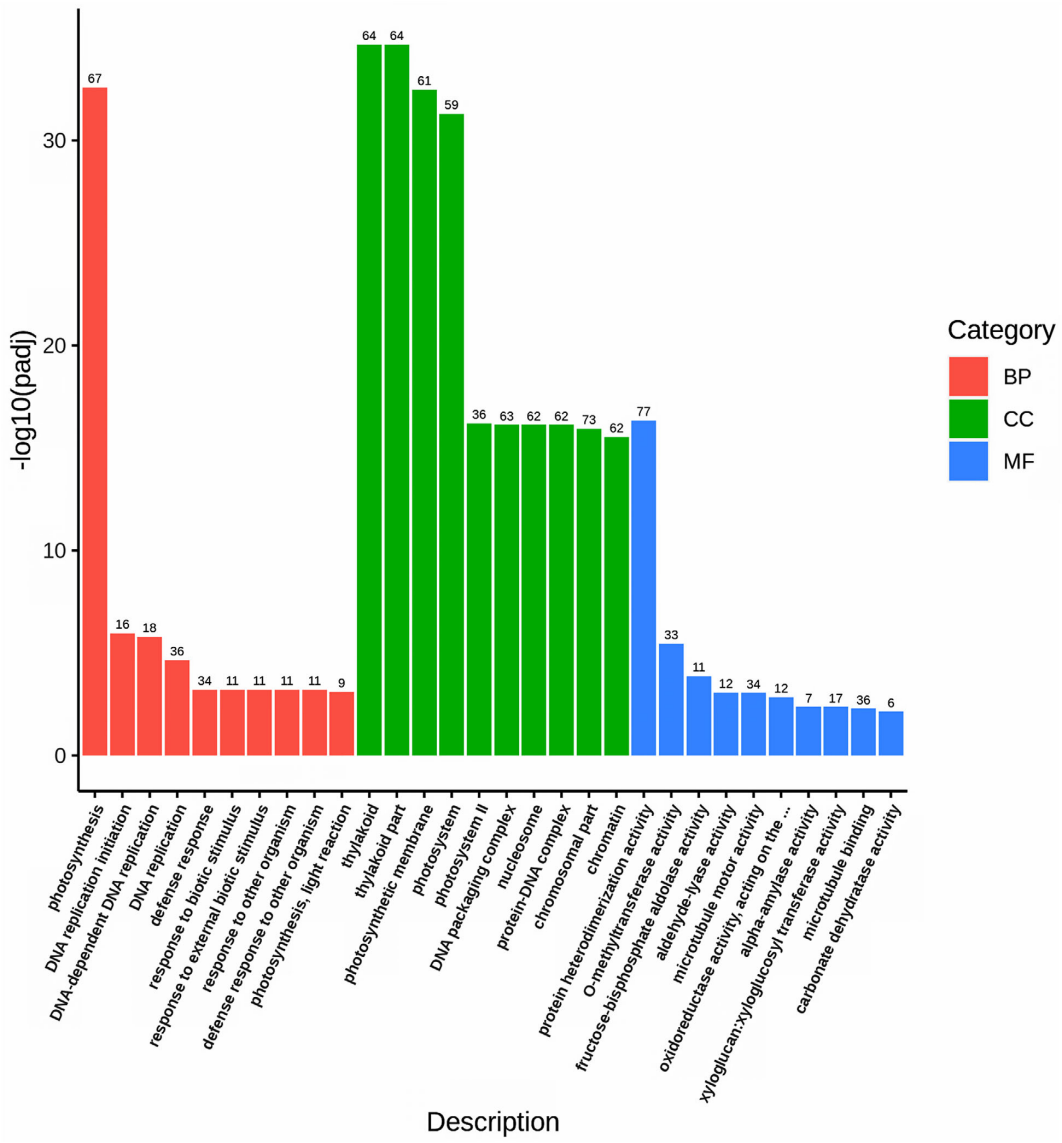
GO enrichment analysis histogram showing overrepresented terms. The abscissa represents a GO term, and the ordinate is GO term's significance of enrichment expressed as–log10(*p*adj). Different colours represent different functional categories.

Application of GO analysis to identify DEGs belonging to biological processes (BP), molecular functions (MF), and cellular components (CC) aspects, resulted in three clearly over‐represented terms: photosynthesis (BP), thylakoid (CC), and protein heterodimerization activity (MF) (Fig. 6). While the first two confirm essential vulnerability of photosynthetic reactions and structural elements to biotic stressors, the third term shows the importance of protein complexes formation in central parts of plant pathogenesis, including regulation of defence responses (Zhang *et al*. 2012).

The GO enrichment analysis to identify overrepresented categories among upregulated genes showed that those mainly belonged to DNA replication processes and responses to stress, while enrichment of downregulated genes in the ‘S’ phenotype identified overrepresentation of photosynthesis‐related genes and genes involved in energy metabolism (Tables S7 and S8). KEGG analysis, in general, confirmed these observations: key genes involved in photosynthesis were downregulated in the ‘S’ phenotype as compared to the ‘A’ phenotype (Figure S9).

To further interpret gene expression data by estimating correlation between gene sets and the defined biological states/phenotypes (‘A’ and ‘S’), a GSEA test was performed. GSEA showed 89 upregulated gene sets in the ‘S’ phenotype, of which 49 sets were significantly enriched at FDR < 25%, and 23 sets were significantly enriched at nominal <1% (Table S9). In the ‘A’ phenotype, GSEA revealed 49 sets, of which 37 were significantly enriched at FDR <25% and 20 gene sets were significantly enriched at nominal <1% (Table S10).

When using KEGG analysis, the top ten enriched sets in phenotype ‘S’ were related to DNA replication, transporter proteins, chromatin remodelling, ribosome biogenesis, and recombination, while the top ten enriched sets in phenotype ‘A’ were mostly associated with photosynthesis (Tables S9 and S10). A heat map of the top 50 DEGs in each phenotype and ranked gene list correlation profile are shown in Table S10.

**Fig. 7:**
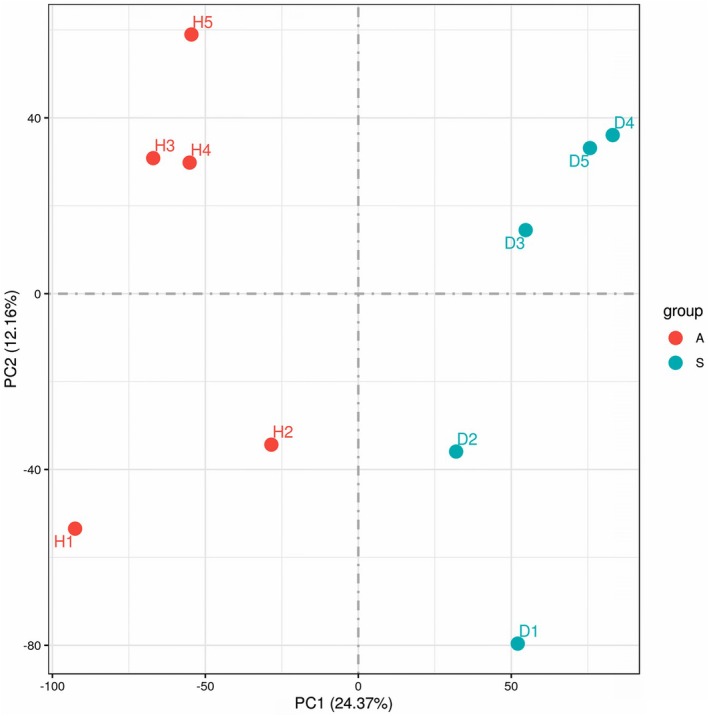
Principal components analysis (PCA) showing intergroup and intragroup differences. The PCA was performed based on the gene expression value (FPKM) of all samples.

A PCA analysis of the gene expression value (FPKM) was performed to evaluate intergroup and intragroup differences. PCA showed that samples of different phenotypes were clearly dispersed, while samples within each phenotype were generally clustered, indicating diverse expression patterns between symptomatic and asymptomatic plants and an analogous pattern within each group (Fig. 7). Interestingly, samples collected from conventional cultivars spaced apart from the Roundup ready alfalfa within each of the groups (asymptomatic and symptomatic). Genetically modified Roundup ready alfalfa is resistant to the herbicide glyphosate, which may affect composition of the pathobiome. However, regardless of the differences between pathobiomes of conventional and Roundup ready samples, it is evident that two main groups (asymptomatic and symptomatic) are clearly dispersed from each other, while the samples within groups clustered together. In other words, expression patterns between asymptomatic and symptomatic plants of Roundup ready cultivars diverge in the same manner as in the conventional cultivars used in the study.

Based on the analyses described above, two shortlists of genes were compiled including (1) genes upregulated in the symptomatic phenotype and likely involved in broad alfalfa responses to the collective pathobiome, and (2) genes highly expressed in asymptomatic phenotype and potentially responsible for alfalfa tolerance to the collective pathobiome (Table S1).

#### Interactions between proteins encoded by DEGs

To further understand reasons behind tolerance to multi‐pathogenic infections, we analysed protein–protein interactions (PPI) in asymptomatic plants. The physical interactions between proteins, although transient and dynamic, can affect many biological processes including plant defence and stress responses (Zhang *et al*. 2010; Struk *et al*. 2018).

The PPI analysis showed that many proteins encoded by DEGs upregulated in symptomless plants (i.e., downregulated in diseased plants) and clustered together in the network, belong to the GO terms associated with photosynthesis (Figure S11). This implies that the proposed physical contacts between proteins are specific and have a particular biological meaning, which, in this case, would presumably be involvement in host–pathogen interactions and immune response (Figure S11 and Table S11). Among the largest group of ~25 closely interacting proteins were light‐harvesting chlorophyll *a*‐*b* binding proteins, involved in abscisic acid signalling (Liu *et al*. 2013); Photosystem II reaction center W protein, one of the central members of photosynthetic machinery (Rhee *et al*. 1998); Photosystem I reaction center subunit psaK, regulating light harvesting under stress (Amunts *et al*. 2009); Photosystem I reaction center subunit IV, essential for photosynthesis, among others (Figure S11).

## DISCUSSION

In this work, we applied a novel “field host genomics” approach toward the study of alfalfa transcriptomic responses to a collective field pathobiome represented by viral, bacterial, and fungal organisms. The approach aimed at the evaluation of genetic responses to a collective pathobiome in the natural field environment, rather than to a single pathogen under controlled experimental conditions. Presumably, this strategy can offer insights into the genetic basis of host resistance to multi‐pathogenic infections in natural ecosystems and help to identify plants with tolerant genotypes adapted to field pathobiome that could be used in breeding programs.

Identified pathogenic species previously reported as pathogenic on alfalfa included AMV, PeSV, BLRV, *Pseudomonas viridiflavia*, *Agrobacterium rubi*, *Acidovorax* sp., *Alternaria* sp., *Stemphylium* sp., *Ascochyta medicaginicola*, *Cladosporium herbarum*, *Claviceps purpurea* and other microorganisms. Plants were also infected with cryptic and not yet fully characterized viruses, beneficial and non‐pathogenic bacterial and fungal species.

Notably, the microbiome of asymptomatic and symptomatic alfalfa field plants was found to be comparable, rather than rigorously distinct, which was hypothesized based on the presence or absence of the disease symptoms. This fact suggests that healthy‐appearing plants may exhibit tolerance to the multi‐pathogenic infections and are able to reduce the effect of the pathobiome on their overall fitness (Pagan & Garcia‐Arenal 2018). However, judging from the sequencing data, pathogens' multiplication process was not limited in “healthy” plants, implying that resistance mechanisms (as opposed to tolerance) are less likely to play a pivotal role in lack of symptom expression.

Various reasons can potentially contribute to alfalfa tolerance to the pathobiome, considering that asymptomatic and symptomatic plants were collected from the same fields seeded with the same varieties, and growing under the same environment:

1. Tolerance observed in asymptomatic plants appears to be under genetic control, as they had uniquely expressed genes silent in symptomatic plants, and the number of DEGs and their expression levels differed from the diseased phenotype.
2. Beneficial microorganisms found in asymptomatic plants can help plants resist pathogens. It is known that beneficial microbes can trigger plant immune responses (Van Wees *et al*. 2008). Asymptomatic plants contained beneficial microorganisms (Table S3), that could potentially contribute to the activation of the plant immune responses.
3. Tolerant plants maintained at the upregulated level specific cellular processes, classified into enriched GO categories *photosynthesis*, *thylakoid*, *photosynthetic membrane*, and *oxidoreductase activity*. In other words, robust response of chloroplasts to multi‐pathogenic infection may be critical for the establishment of tolerance as defence against microbial attack required energy provided by photosynthesis (Serrano *et al*. 2016). Chloroplasts play essential roles in plant defence reactions (Kachroo *et al*. 2021), and the majority of the disease symptoms observed in the field directly affected chloroplasts. Elevated expression of the genes encoding chloroplastic proteins in the ‘A’ phenotype can be associated not only with the absence of the disease symptomatology (i.e., undamaged chloroplasts), but also with effective immune response to pathogens attack that is, increased tolerance to the field pathobiome. This conclusion is supported by analysis of PPI in the asymptomatic plants.
4. Increased photosynthetic activity in tolerant plants resulted mainly from overexpression of genes encoding proteins catalysing carbon fixation (e.g., MS_gene62198), structural components of chloroplasts (e.g., MS_gene31538) and proteins of Photosystems I and II (e.g., MS_gene03163 and MS_gene75549).
5. In addition to genes involved in photosynthesis, genes encoding proteins localized in chloroplasts and known to modulate defence responses were upregulated in tolerant plants, such as calcium‐sensing receptor (MS_gene044510), thylakoid formation 1 protein (MS_gene31538), and light‐harvesting complex‐like protein OHP1 (MS_gene89341).
6. Genes not involved in photosynthesis also participated in tolerant response. Among these were HAD, regulating phosphate homeostasis during phosphorus (Pi) deficiency stress (Du *et al*. 2021); 40S ribosomal protein S3a with many roles including host–pathogen interactions (Gao & Hardwidge 2011); disease resistance proteins; plasma membrane ATPase participating in plant immune responses (Elmore & Coaker 2011), WRKY TFs, known for their role in plant immunity (Pandey and Somssich, 2009).

**Fig. 8:**
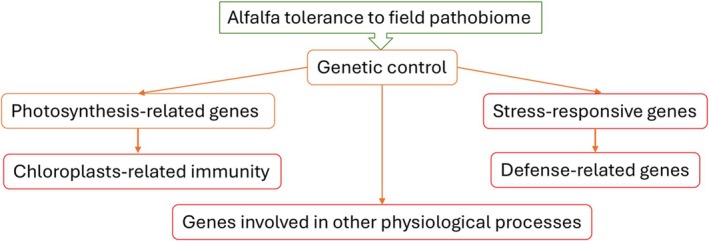
A simplified diagram, illustrating the contribution of different pathways toward alfalfa tolerance to the field pathobiome.

A simplified diagram, illustrating the contribution of different pathways toward alfalfa tolerance to the field pathobiome is shown in Fig. 8. While the combination of all relevant factors is important, chloroplasts appear to play a major role in promoting alfalfa's ability to reduce the effect of multi‐pathogenic infections on the plant fitness. Knowing the genes controlling this important and likely polygenic trait can provide an alternative to host resistance‐based management practices of plant diseases (Jeger 2023).

## AUTHOR CONTRIBUTIONS

**LGN:** Concept, data analysis, and first draft of the manuscript. **BMI:** Survey, sample collection, and evaluation. **SG:** Bioinformatics and data analysis. **OAP:** Wet lab and data analysis. All authors contributed to the editing of the manuscript and approved it for publication.

## CONFLICT OF INTEREST

The authors declare that the research was conducted in the absence of any commercial or financial relationships that could be construed as a potential conflict of interest.

## Supporting information


**Figure S1.** Workflow of the bioinformatics analysis.

**Figure S2.** Taxonomic abundance cluster heatmap of bacterial species. The heatmap shows whether samples with similar processing are clustered or not, while the similarity and differences between the samples can also be observed.

**Figure S3.** Alpha diversity of microbial communities in each group. Horizontal axis represents groups, while vertical axis represents the corresponding alpha diversity index value. Group G1.2, five asymptomatic plants; group G2.2, five symptomatic plants.

**Figure S4.** Beta diversity heatmap. Numbers in grids are dissimilarity coefficient between samples. Two numbers in the same grid represent weighted and unweighted Unifrac distance, respectively.

**Figure S5.** Taxonomic cluster heatmap of fungal species.

**Figure S6.** Alpha diversity of the observed fungal species in each group. Group G1 composed of five asymptomatic plants; group G2– of five symptomatic plants.

**Figure S7.** Beta diversity heatmap showing diversity of fungal communities between different samples. Numbers in grids are dissimilarity coefficient between samples. Two numbers in the same grid represent weighted and unweighted Unifrac distance, respectively.

**Figure S8.** Heatmap depicting DEGs in both phenotypes.

**Figure S9.** KEGG pathway depicting downregulation of photosynthesis‐related genes in symptomatic phenotype.

**Figure S10.** Heat map depicting top 50 DEGs in each phenotype.

**Figure S11.** Protein–protein interaction (PPI) network. The network was constructed by searching the protein interaction database STRING (https://string‐db.org) and visualized using Cytoscape software v. 3.10.3. For GO enrichment, all connected nodes in the main network were selected and STRING functional enrichment option applied. A, Overrepresented GO terms shown in green. B, Non‐redundant GO terms. C, Genes encoding interacting proteins (Table S11).

**Table S1.** Plant samples, RNA‐seq metrics and reads, and genes differentially expressed in asymptomatic and symptomatic plants.

**Table S2.** Viruses identified in symptomatic (D1, D2, D3, D4, and D5) and asymptomatic (H1, H2, H3, H4, and H5) plant samples.

**Table S3.** Bacterial and fungal species identified in all samples.

**Table S4.** DEGs identified in symptomatic (‘S’) and asymptomatic (‘A’) phenotypes.

**Table S5.** Upregulated DEGs identified in symptomatic (‘S’) phenotype.

**Table S6.** Downregulated DEGs identified in symptomatic (‘S’) phenotype.

**Table S7.** Overrepresented GO categories among upregulated genes of the symptomatic (‘S’) phenotype.

**Table S8.** Overrepresented GO categories among downregulated genes of phenotype ‘S’ (symptomatic).

**Table S9.** Gene sets enriched in phenotype ‘A’ (asymptomatic).

**Table S10.** Gene sets enriched in phenotype ‘S’(symptomatic).

**Table S11.** Interactions between proteins encoded by genes downregulated in the symptomatic phenotype (upregulated in asymptomatic phenotype ‘A’).

## Data Availability

The datasets have been submitted to the NCBI's Sequence Read Archive (SRA) under the Submission ID: SUB14892281 and the BioProject accession ID: PRJNA1191001.
