# Supplementary figures and images for: Alfalfa transcriptomic responses to the field pathobiome
Source: Plant Biol (Stuttg). 2025 Apr 15;27(4):492–503. doi: 10.1111/plb.70021 (PMC12096062; doi:10.1111/plb.70021)

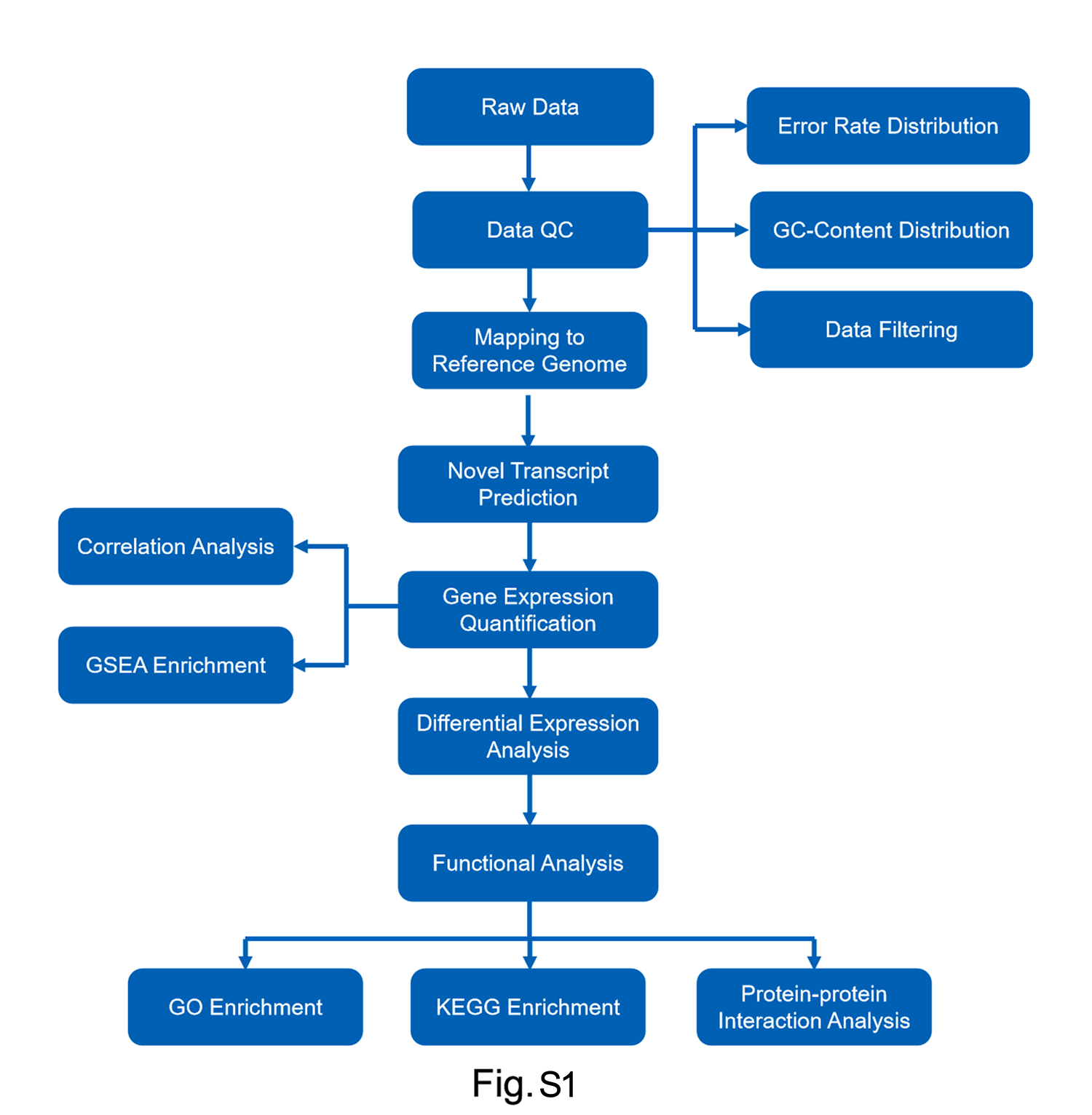

Supplement: Supplementary file 1 — Figure S1. Workflow of the bioinformatics analysis. [file PLB-27-492-s001.tif]

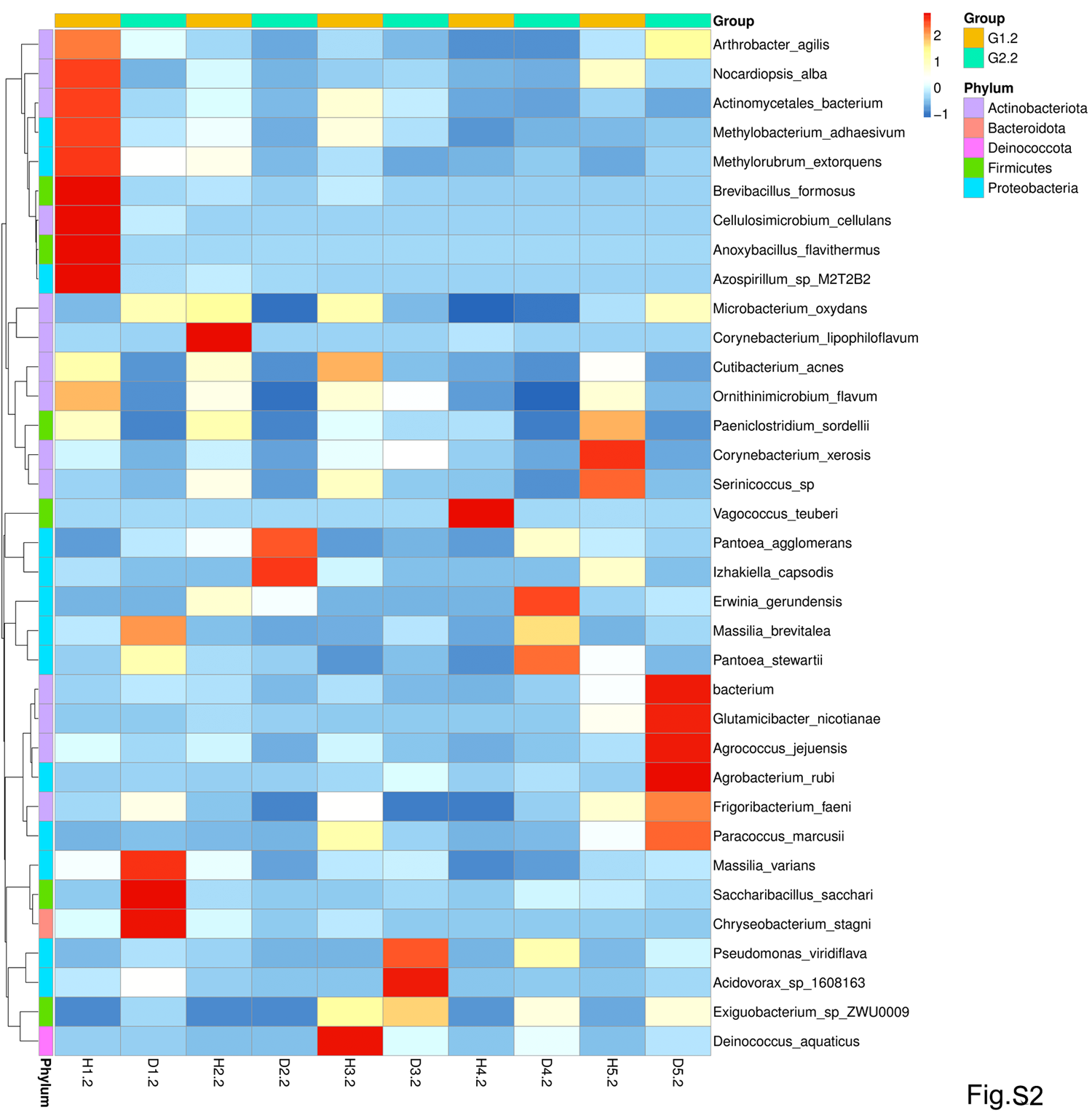

Supplement: Supplementary file 2 — Figure S2. Taxonomic abundance cluster heatmap of bacterial species. The heatmap shows whether samples with similar processing are clustered or not, while the similarity and differences between the samples can also be observed. [file PLB-27-492-s015.tif]

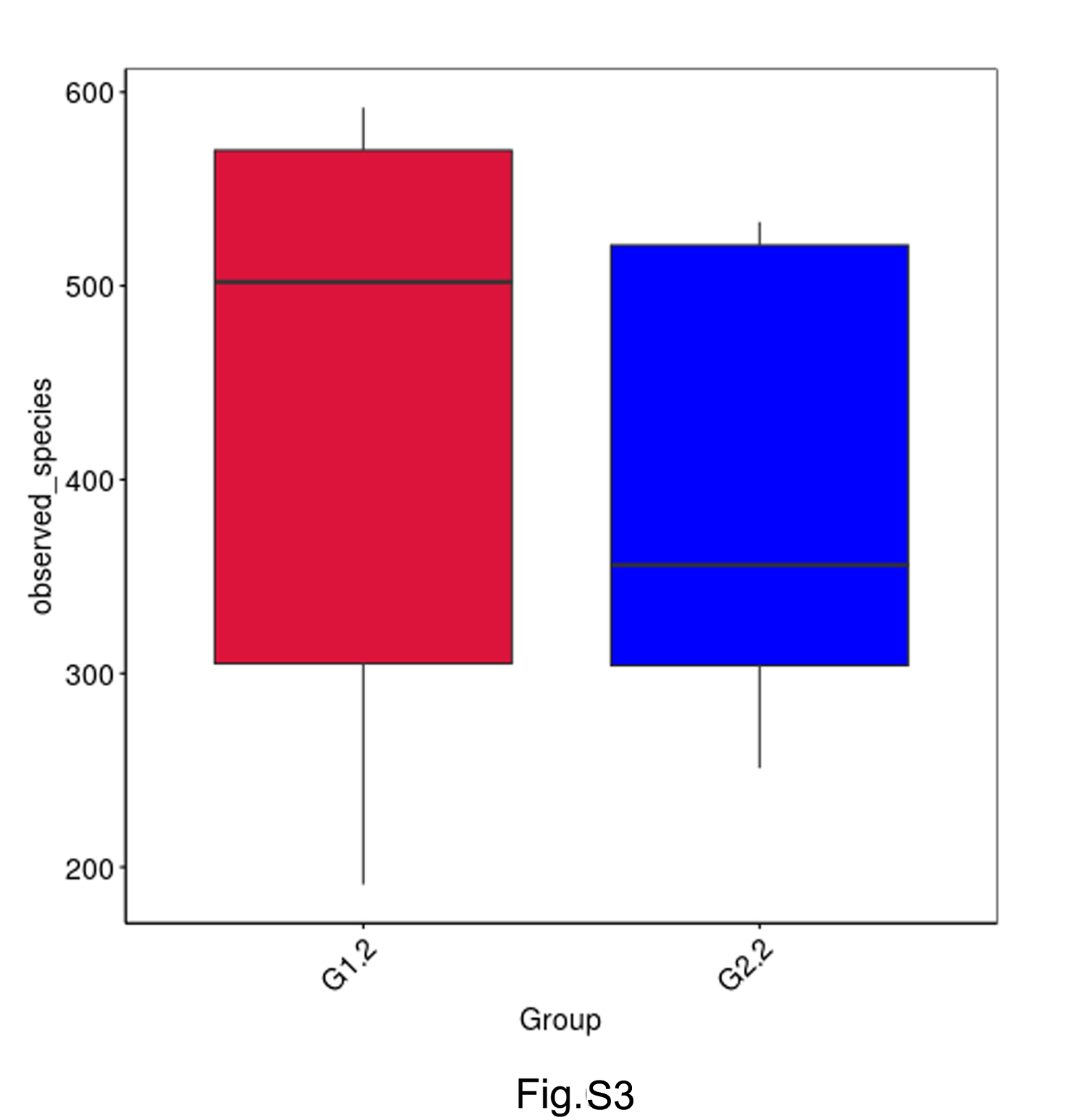

Supplement: Supplementary file 3 — Figure S3. Alpha diversity of microbial communities in each group. Horizontal axis represents groups, while vertical axis represents the corresponding alpha diversity index value. Group G1.2, five asymptomatic plants; group G2.2, five symptomatic plants. [file PLB-27-492-s008.tif]

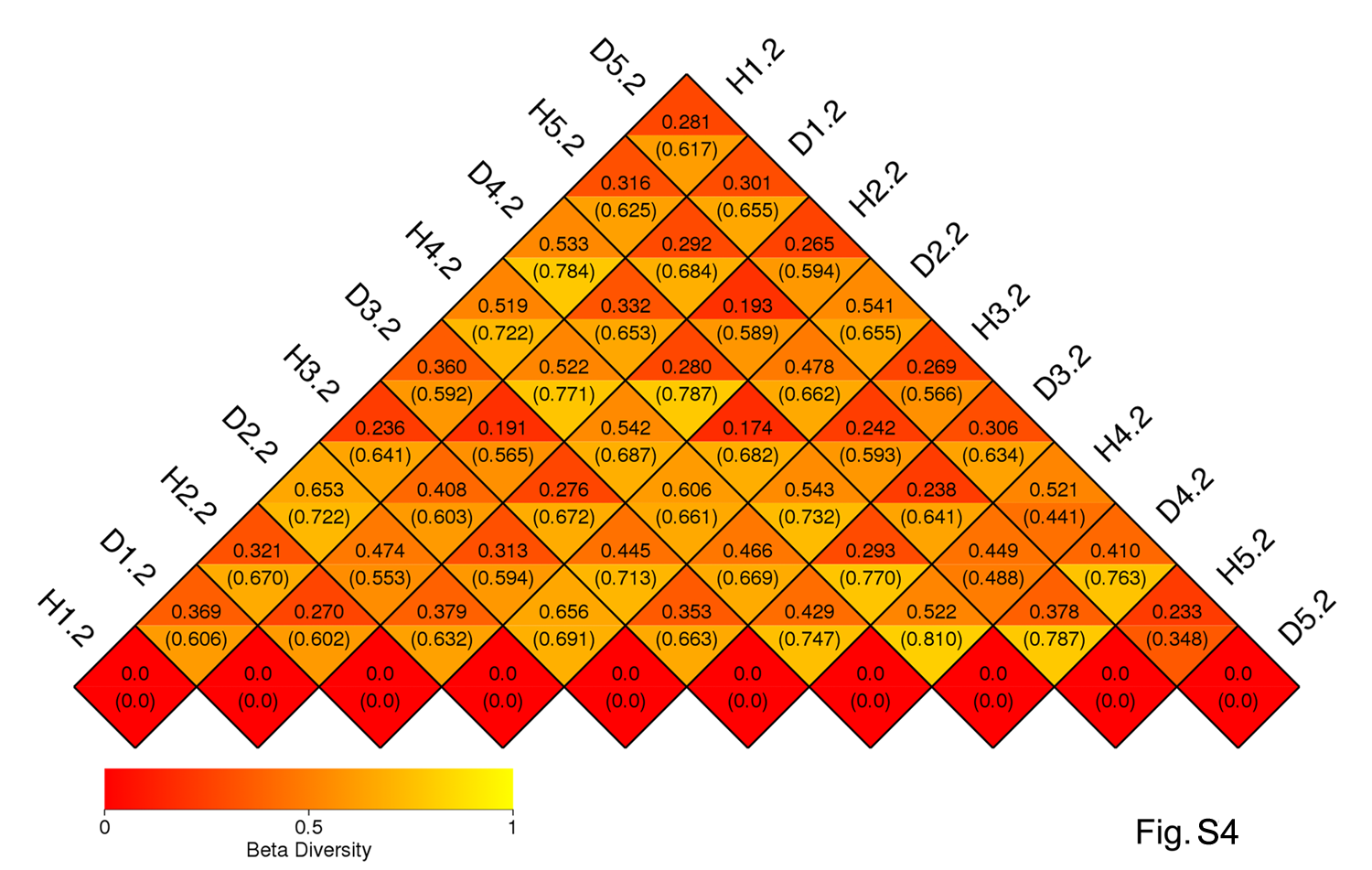

Supplement: Supplementary file 4 — Figure S4. Beta diversity heatmap. Numbers in grids are dissimilarity coefficient between samples. Two numbers in the same grid represent weighted and unweighted Unifrac distance, respectively. [file PLB-27-492-s004.tif]

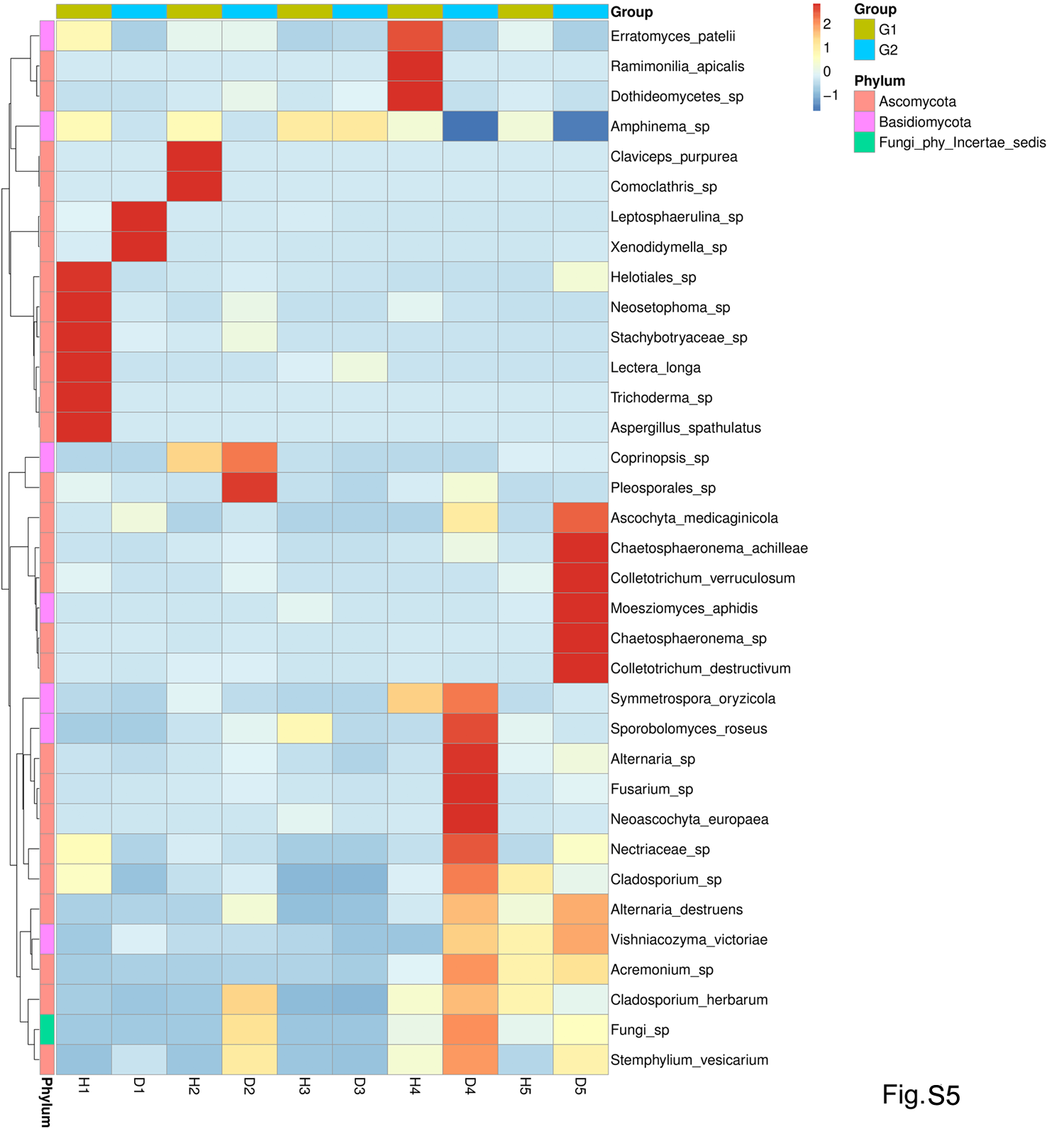

Supplement: Supplementary file 5 — Figure S5. Taxonomic cluster heatmap of fungal species. [file PLB-27-492-s009.tif]

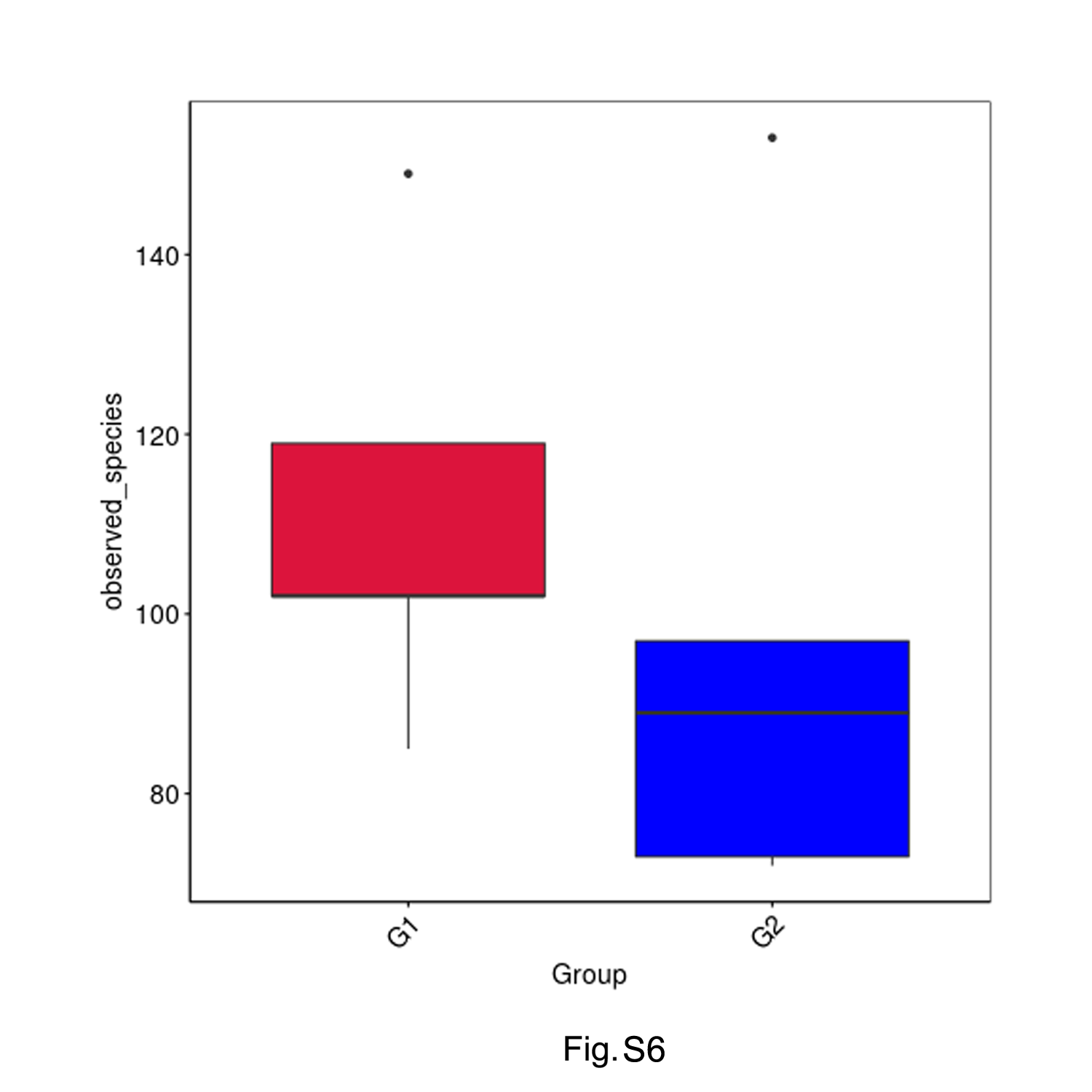

Supplement: Supplementary file 6 — Figure S6. Alpha diversity of the observed fungal species in each group. Group G1 composed of five asymptomatic plants; group G2– of five symptomatic plants. [file PLB-27-492-s010.tif]

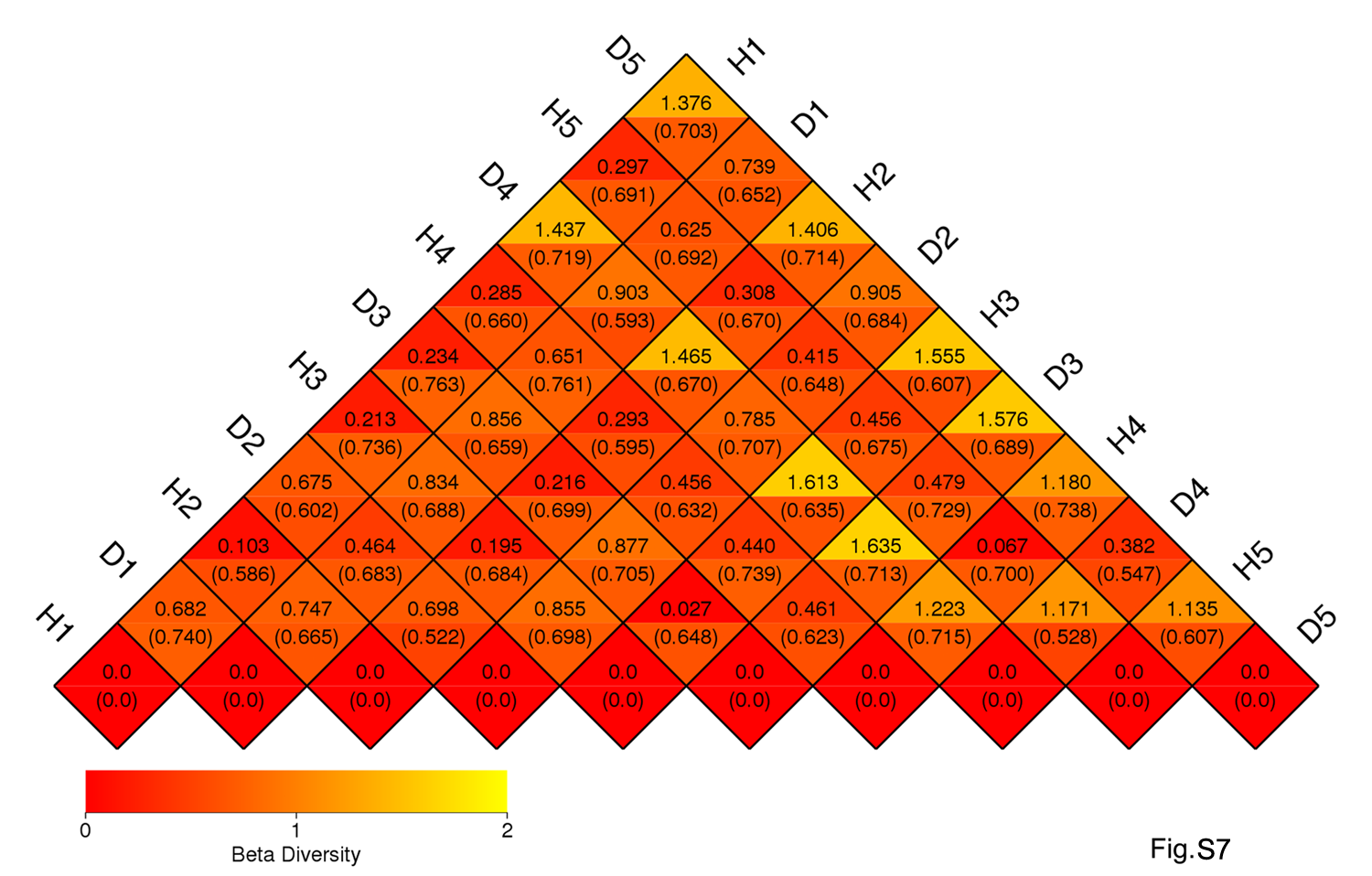

Supplement: Supplementary file 7 — Figure S7. Beta diversity heatmap showing diversity of fungal communities between different samples. Numbers in grids are dissimilarity coefficient between samples. Two numbers in the same grid represent weighted and unweighted Unifrac distance, respectively. [file PLB-27-492-s021.tif]

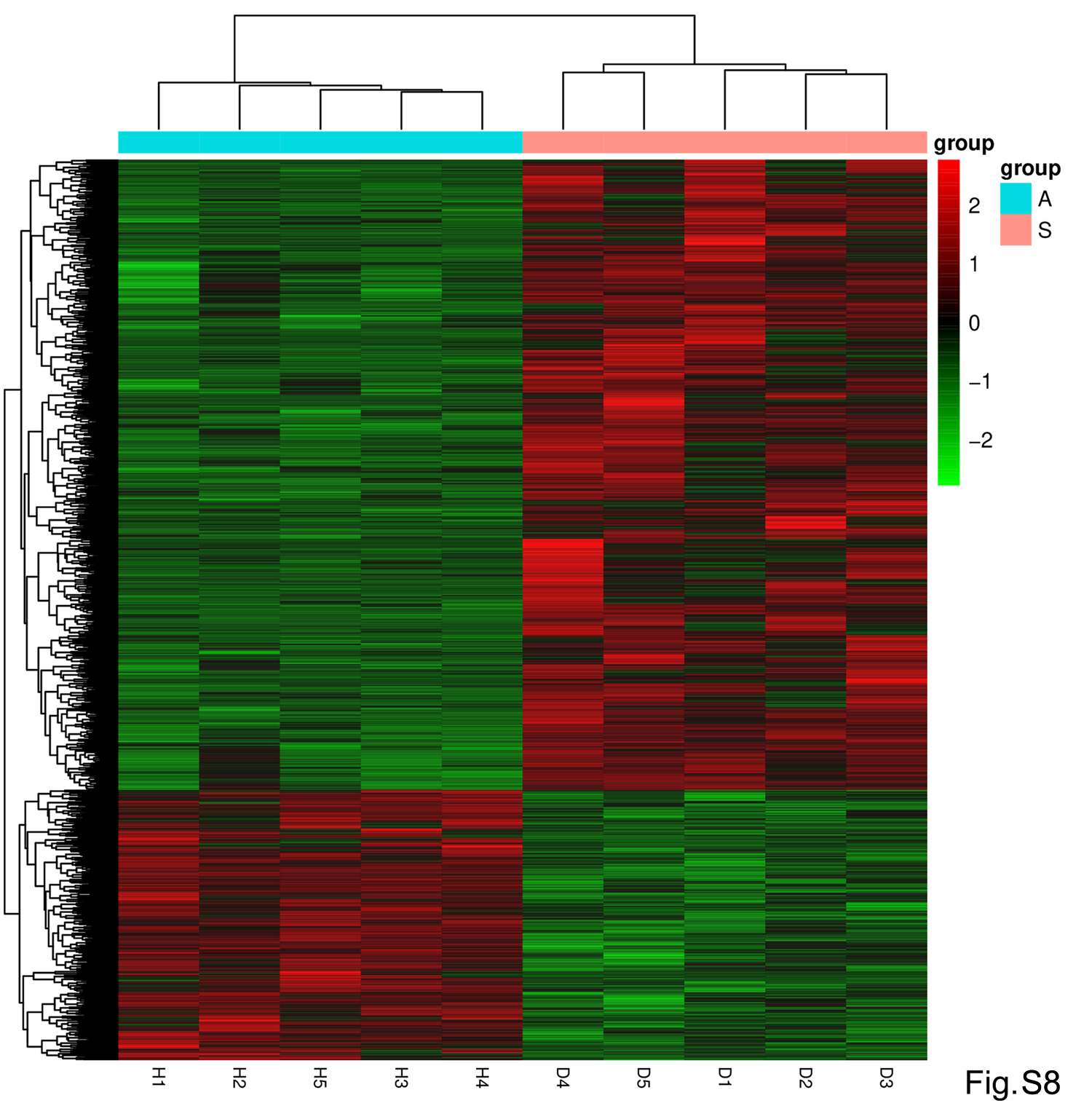

Supplement: Supplementary file 8 — Figure S8. Heatmap depicting DEGs in both phenotypes. [file PLB-27-492-s006.tif]

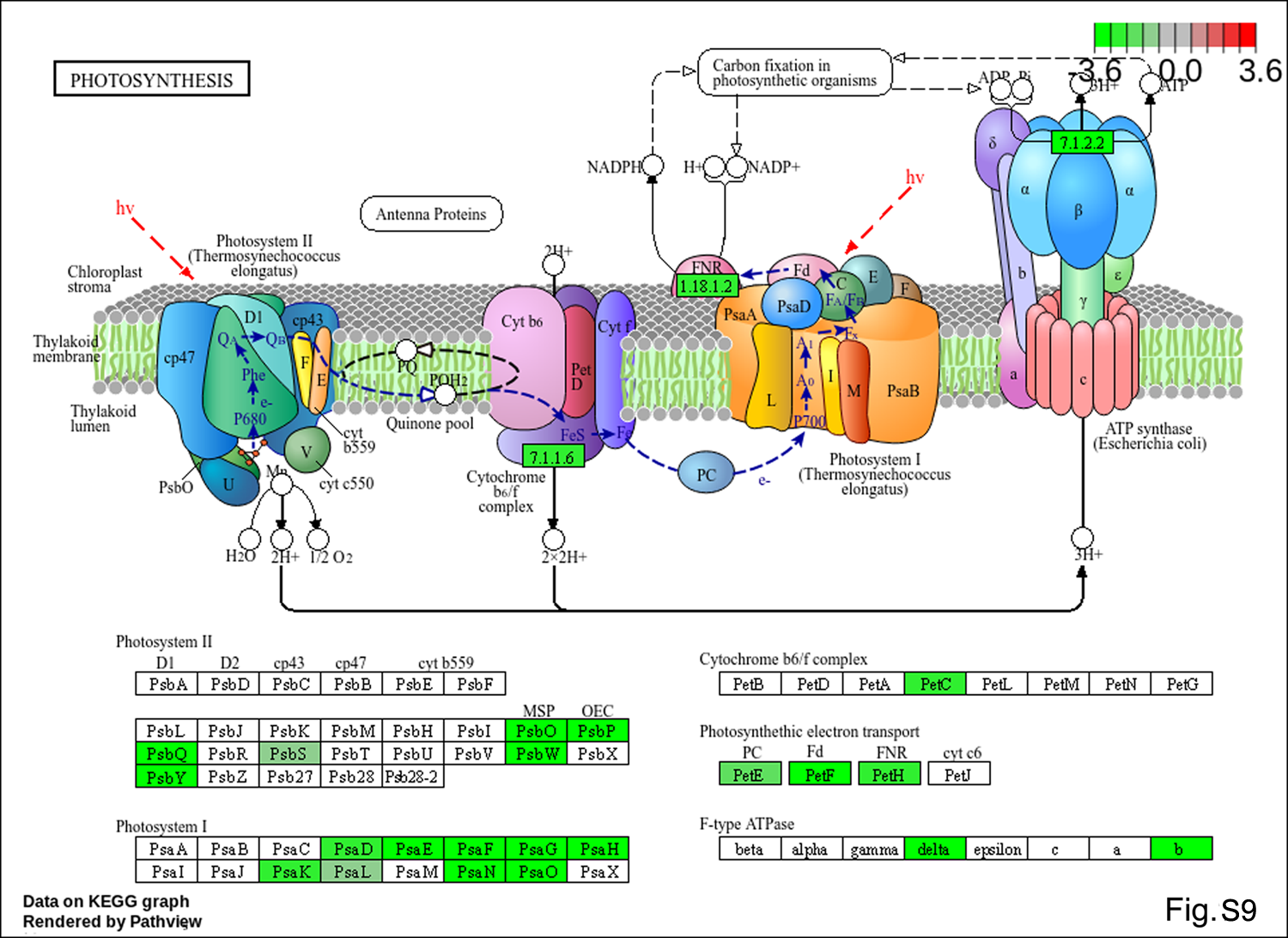

Supplement: Supplementary file 9 — Figure S9. KEGG pathway depicting downregulation of photosynthesis‐related genes in symptomatic phenotype. [file PLB-27-492-s011.tif]

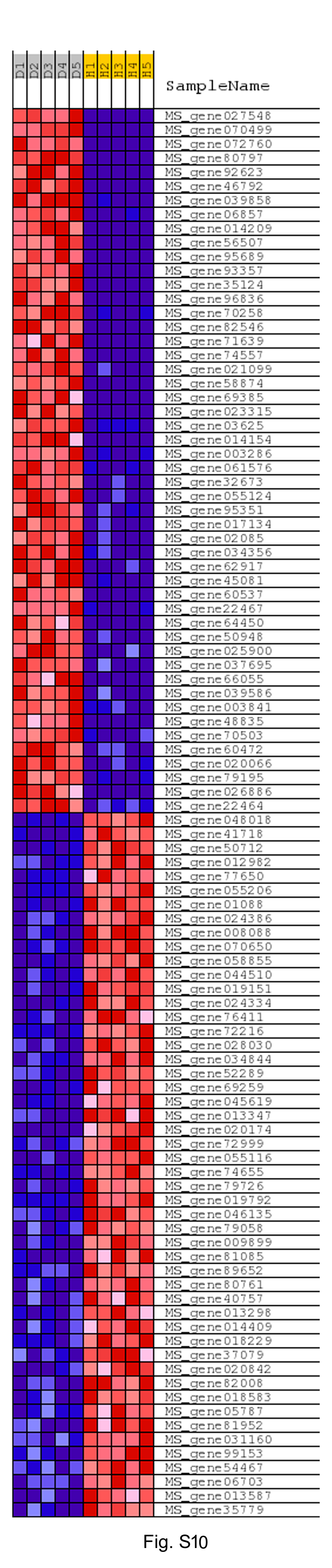

Supplement: Supplementary file 10 — Figure S10. Heat map depicting top 50 DEGs in each phenotype. [file PLB-27-492-s013.tif]

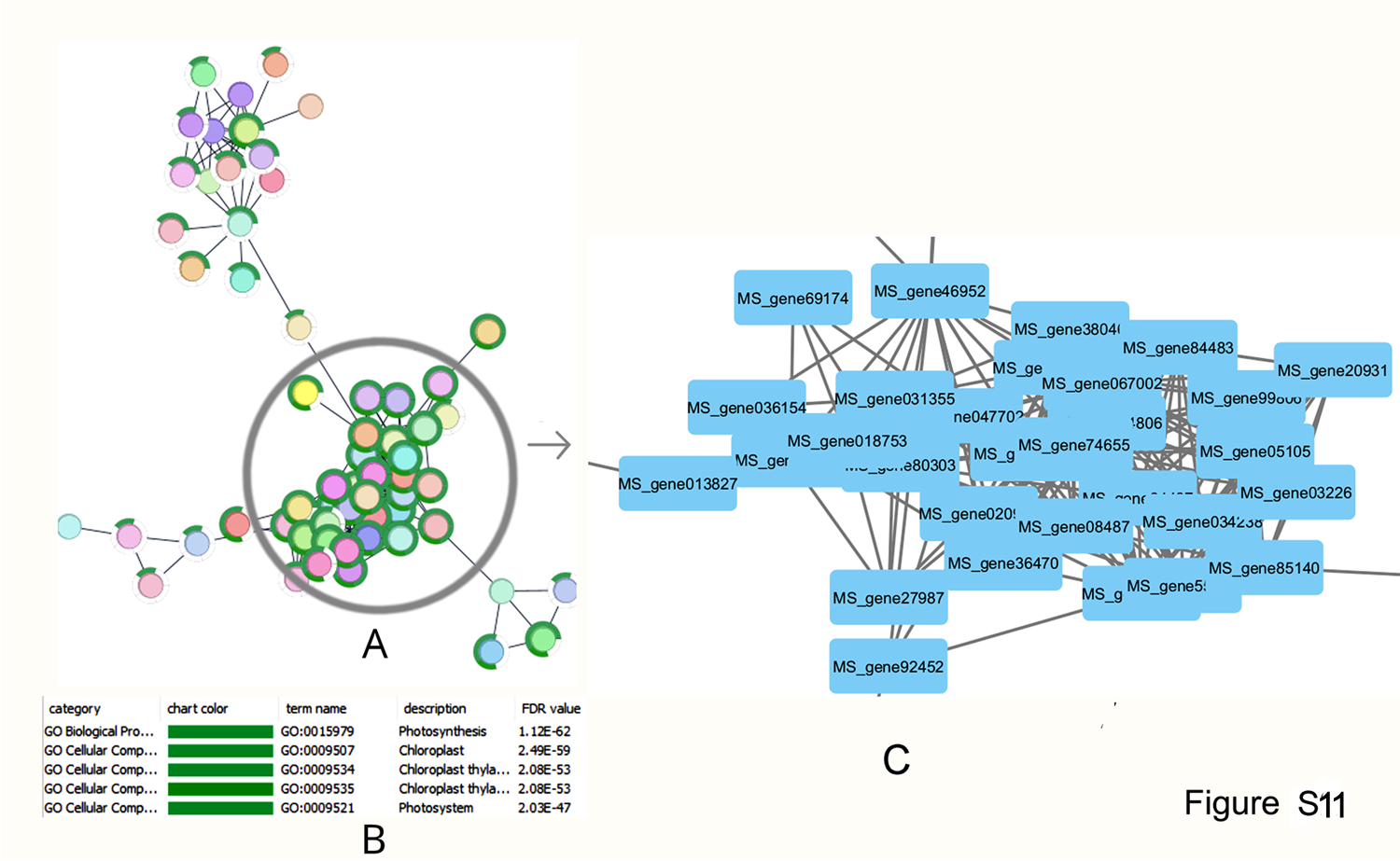

Supplement: Supplementary file 11 — Figure S11. Protein–protein interaction (PPI) network. The network was constructed by searching the protein interaction database STRING (https://string‐db.org) and visualized using Cytoscape software v. 3.10.3. For GO enrichment, all connected nodes in the main network were selected and STRING functional enrichment option applied. A, Overrepresented GO terms shown in green. B, Non‐redundant GO terms. C, Genes encoding interacting proteins (Table S11). [file PLB-27-492-s007.tif]
